# In Situ Carbon-Confined MoSe_2_ Catalyst with Heterojunction for Highly Selective CO_2_ Hydrogenation to Methanol

**DOI:** 10.3390/molecules29102186

**Published:** 2024-05-08

**Authors:** Yanyang Sun, Linfei Xiao, Wei Wu

**Affiliations:** National Center for International Research on Catalytic Technology, School of Chemistry and Material Sciences, Heilongjiang University, Harbin 150080, China; sunyanyang99@163.com

**Keywords:** MoSe_2_@C, CO_2_, methanol, hydrogenation

## Abstract

The synthesis of methanol from CO_2_ hydrogenation is an effective measure to deal with global climate change and an important route for the chemical fixation of CO_2_. In this work, carbon-confined MoSe_2_ (MoSe_2_@C) catalysts were prepared by in situ pyrolysis using glucose as a carbon source. The physico-chemical properties and catalytic performance of CO_2_ hydrogenation to yield methanol were compared with MoSe_2_ and MoSe_2_/C. The results of the structure characterization showed MoSe_2_ displayed few layers and a small particle size. Owing to the synergistic effect of the Mo_2_C-MoSe_2_ heterojunction and in situ carbon doping, MoSe_2_@C with a suitable C/Mo mole ratio in the precursor showed excellent catalytic performance in the synthesis of methanol from CO_2_ hydrogenation. Under the optimal catalyst MoSe_2_@C-55, the selectivity of methanol reached 93.7% at a 9.7% conversion of CO_2_ under optimized reaction conditions, and its catalytic performance was maintained without deactivation during a continuous reaction of 100 h. In situ diffuse infrared Fourier transform spectroscopy studies suggested that formate and CO were the key intermediates in CO_2_ hydrogenation to methanol.

## 1. Introduction

A large amount of CO_2_ is emitted when extravagant fossil fuels (coal, oil and natural gas) are consumed, which causes a series of climate problems [1,2]. In view of the current situation, in order to reduce CO_2_ emissions, carbon capture and storage (CCS) and carbon capture and utilization (CCU) are currently the most effective means [3]. However, the high cost and energy consumption of desorption, compression and storage of captured CO_2_ greatly limit the large-scale application of CCS. CCU can achieve efficient CO_2_ recovery and bring certain economic benefits, and it is considered a promising technology to reduce CO_2_ emissions. As a raw material, CO_2_ can be converted to fuel and value-added chemicals [4,5]. On the one hand, it can reduce the pressure of the fossil resource shortage; on the other hand, it can bring huge economic benefits. Therefore, the chemical utilization of CO_2_ to produce value-added chemicals has become one of the hot topics for using a non-toxic, inexpensive, abundant and renewable source of carbon [6,7,8,9]. Among the CO_2_ utilization routes, synthesizing methanol has received extensive attention [10,11]. Methanol is water-soluble, non-toxic, harmless and easy to store and transport, and it is known as the simplest ‘alcohol’, so it has become one of the most important platform compounds in the chemical industry [12]. Methanol can be miscible with water and a variety of organic matter, serving as an alternative clean fuel, liquid organic hydrogen carrier and common organic solvent [12,13,14]. It can also be used to produce olefins, aromatic hydrocarbons, alkanes and other bulk chemicals [15,16]. At present, the global production of methanol is 110 million metric tons [17].

For the hydrogenation of CO_2_ to methanol, the utilization of copper-based catalysts has garnered significant interest. The main active substances of copper-based catalysts in over 60% of related reports are Cu-ZnO composite materials [18]. However, two disadvantages hinder the widespread use of copper-based catalysts. One is that the generation of water in the reaction aggravates the sintering of the active phase, resulting in poor stability of the catalyst, and the other is that the selectivity of methanol is limited due to the reverse water gas reaction [19,20]. Compared with copper-based catalysts, noble metal-based catalysts exhibit excellent stability, anti-sintering and anti-poisoning properties and can be used as an effective alternative to copper-based catalysts. Although noble metal catalysts show a high ability to activate hydrogen and promote hydrogenation, the low selectivity of methanol is yielded due to its weak interaction with CO_2_ in the reaction process. Meanwhile, its low abundance and high cost also limit its large-scale application in CO_2_ hydrogenation to methanol. Recently, metal oxide catalysts such as In_2_O_3_ and Zn-Zr solid solutions have attracted attention [21]. Due to the formation of oxygen vacancies under reaction conditions of 330 °C and 5 MPa, In_2_O_3_ provided 100% methanol selectivity in CO_2_ hydrogenation, but it could be reduced to In^0^ metal, which caused the deactivation during the reaction [22]. Under industrial conditions, the ZnO-ZrO_2_ solid solution catalyst demonstrated the capability to facilitate the hydrogenation of CO_2_ to methanol [23]. The space–time yield of methanol was as high as 7.75 mol·kg^−1^·h^−1^ with an 87.0% selectivity of methanol, and excellent stability was shown in the presence of SO_2_ and H_2_S. However, shortcomings are shown in the harsh reaction conditions, poor activity and high energy consumption. Therefore, it is still a challenge to develop highly effective non-precious metal catalysts for CO_2_ hydrogenation to form methanol under relatively mild conditions.

Because of their large surface area and large amounts of coordination-unsaturated surface atoms, two-dimensional layered Mo-based catalysts have attracted widespread attention [24,25]. Due to its similar electronic structure to the noble metal Pt, molybdenum carbide displays excellent catalytic performance for hydrogenation and has been applied in hydrodeoxygenation [26,27] and hydrodenitrogenation [28]. In 1992, molybdenum carbide was used as a catalyst for the hydrogenation of CO_2_, resulting in a methanol selectivity of only 17%, with methane and CO being the predominant byproducts [29]. DFT results suggested that enhancing the adsorption of CO_2_ on the catalyst surface was beneficial for inhibiting the complete decomposition of CO_2_, thus improving the selectivity of methanol [30]. In our previous work, N,S-doped carbon-confined molybdenum carbide catalysts were synthesized by in situ pyrolysis carbonization [31]. The basic sites generated from N and S in the in situ pyrolysis carbonization increased the adsorption amount of CO_2_. The selectivity of methanol yielded 90% with a CO_2_ conversion of 20%. In this catalyst, nano-sized MoS_2_ was also generated in the pyrolysis process, which was a key factor in its excellent catalytic performance. On the one hand, in the presence of MoS_2_, CO_2_ was adsorbed and dissociated at sulfur vacancies; on the other hand, in-plane sulfur vacancies could inhibit the deep hydrogenation of CO_2_ to methane and promote the formation of methanol at low temperatures. More recently, Deng [32] et al. constructed a surface-rich sulfur-vacancy MoS_2_ catalyst and applied it for the hydrogenation of CO_2_ to form methanol, with the methanol selectivity reaching 94.3% with a CO_2_ conversion of 12.5% at 180 °C. MoSe_2_, as a two-dimensional layered material, has stronger metallicity and lower Gibbs free energy than molybdenum sulfide [33], which is more conducive to the adsorption and activation of hydrogen molecules [34]. More importantly, the adsorption capacity of CO_2_ is also better than that of MoS_2_ [35]. However, MoSe_2_ is mainly involved in photocatalysis and electrocatalysis for the hydrogenation of CO_2_, and the main reduction products are methane, carbon monoxide and so on [35]. Qu et al. incorporated K into MoSe_2_ and constructed a K-Mo-Se active phase for catalytic syngas conversion to ethanol. By regulating the K/Mo ratio, the proportion of ethanol in the total alcohol reached 58.7% under optimal reaction conditions [36]. To our best knowledge, there is no report on the use of MoSe_2_ as a catalyst in the thermocatalytic hydrogenation of CO_2_ to produce methanol [37,38]. Therefore, the study of the catalytic behavior of MoSe_2_ is significant for the development of non-noble metal catalysts for CO_2_ hydrogenation to methanol. 

It is well known that, as a typical two-dimensional material, the active sites of MoSe_2_ are also mainly located at its edges and defect sites. Therefore, reducing the layer number and particle size of MoSe_2_ is an effective route to enhance its catalytic performance. Confined MoSe_2_ in carbon materials can effectively prevent the aggregation of MoSe_2_ and improve its catalytic performance [39,40,41]. In this work, MoSe_2_ confined by in situ-formed carbon material (MoSe_2_@C) was prepared by introducing glucose as a carbon source into the precursors using the pyrolysis method and applied to CO_2_ hydrogenation to form methanol in a fixed-bed reactor. The influences of the preparation conditions and the C/Mo ratio in the precursors on the catalytic performance of MoSe_2_@C were investigated, and the reaction conditions were optimized. Under optimal reaction conditions, a high methanol selectivity of 93.7% was reached. MoSe_2_@C also showed excellent catalytic stability during performance evaluation and could be used for at least 100 h without deactivation. 

## 2. Results and Discussion 

### 2.1. Catalyst Characterization 

#### 2.1.1. Structural Characterizations

**Figure 1:**
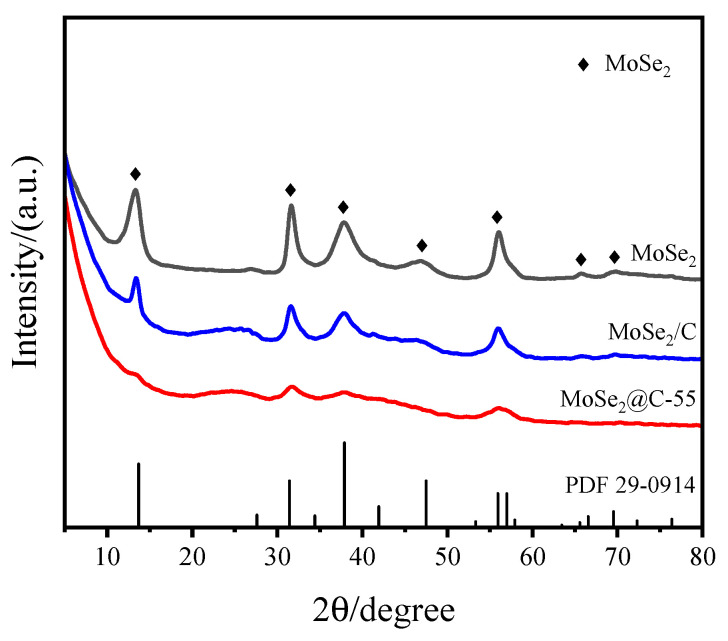
XRD patterns of MoSe_2_, MoSe_2_/C and MoSe_2_@C-55 catalysts.

Firstly, X-ray powder diffraction was employed to detect the crystal structure of the prepared samples, and the results are shown in Figure 1. The characteristic diffraction peaks of the three samples appeared at 2θ = 13.7°, 31.4°, 37.9°, 47.5°, 55.9°, 66.5° and 69.5°, corresponding to the (002), (100), (103), (105), (110), (108) and (203) crystal planes of MoSe_2_ (PDF 29-0914) [42], and no other diffraction peaks were found. These results indicate that the MoSe_2_ crystal phase was prepared. In addition, a slight shift of the (002) crystal plane could be observed, which was attributed to thermal-induced phase segregation [43]. Compared with MoSe_2_, the intensity of the diffraction peaks was weakened, and the half-peak width of the corresponding diffraction peaks increased in the XRD patterns of the MoSe_2_/C sample, indicating that the introduction of a carbon carrier could improve the dispersion of MoSe_2_ and inhibit the growth of MoSe_2_ particles. Additionally, the characteristic diffraction peaks corresponding to the (002) crystal plane of graphite carbon were also found at 2θ = 26°. Compared with the XRD patterns of MoSe_2_ and MoSe_2_/C, the diffraction peak intensity of MoSe_2_@C-55 was the weakest, the half-peak width was the widest, and the characteristic diffraction peak (2θ = 13.7°) corresponding to the (002) crystal plane of MoSe_2_ almost disappeared. These results suggested that the grain size of MoSe_2_ was the smallest and the layer number of MoSe_2_ was the lowest in MoSe_2_@C-55 compared with MoSe_2_ and MoSe_2_/C [44,45]. As a consequence, introducing glucose to the precursor could suppress the growth of MoSe_2_ through the confinement of the in situ generation of carbon material in the pyrolysis process. When the C/Mo ratio in the precursor was raised from 15 to 75, MoSe_2_@C-15, MoSe_2_@C-35 and MoSe_2_@C-75 were prepared. Their XRD patterns are shown in Figure S1. The intensity of the diffraction peak corresponding to MoSe_2_ gradually declined with the enhancement of the carbon content in the precursor, which means that the confinement of the in situ carbon material improved with the increase in carbon content, and the growth of MoSe_2_ particles was repressed, which was beneficial to the dispersion of MoSe_2_ in the carbon material.

**Figure 2:**
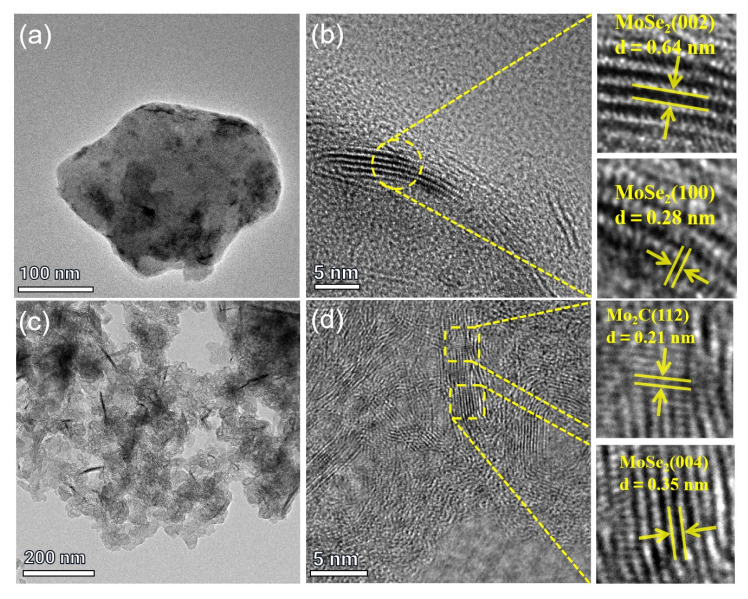
TEM and HRTEM images of MoSe_2_/C and MoSe_2_@C-55 catalysts: (**a**,**b**) MoSe_2_/C; (**c**,**d**) MoSe_2_@C-55.

In order to observe the microstructure and morphology, MoSe_2_/C and MoSe_2_@C-55 were characterized by TEM and HRTEM (Figure 2). The image in Figure 2a shows that MoSe_2_ dispersed on the coconut shell carbon, which resulted in the low intensity of the XRD diffraction peak. However, its distribution was not uniform, and agglomeration was observed, which caused MoSe_2_ to show larger particles and more layers. In Figure 2b (HRTEM), lattice fringes of 0.28 nm can be observed, which is consistent with the interplanar spacing of the (100) of MoSe_2_ [46,47]. Observing the image of MoSe_2_@C-55, as shown in Figure 2c, the agglomeration and accumulation of MoSe_2_ particles were effectively inhibited when glucose was introduced to the precursor. This took place because the carbon materials formed from the in situ pyrolysis of glucose showed an inhibitory effect on the growth of MoSe_2_ particles, which ensured the high dispersion of MoSe_2_. As a result of it, more active sites were exposed, and its catalytic performance improved. As can also be seen from the HRTEM image (Figure 2d), a lattice fringe spacing of 0.35 nm was also observed, which belonged to the (004) of MoSe_2_. In addition, a lattice fringe with a spacing of 0.21 nm was also observed (Figure 2d), which was attributed to the (112) of Mo_2_C [48], indicating that Mo_2_C particles were generated during the preparation of MoSe_2_@C-55. Combined with the fact that the corresponding diffraction peaks of Mo_2_C were not observed in the XRD pattern, it can be inferred that the content of Mo_2_C was low and highly dispersed. In addition, the HRTEM images in Figure 2d show that the presence of Mo_2_C interrupted the continuity of MoSe_2_, causing more edge sites of MoSe_2_ to expose more active sites [49]. This phenomenon indicated that a MoSe_2_-Mo_2_C heterojunction was generated, which would benefit the synergistic effect of MoSe_2_ and Mo_2_C in the catalytic process and improve its catalytic performance [50]. The results in Figure S2a–d show that increasing the content of glucose in the precursor was favorable to improving the dispersion of MoSe_2_, resulting in a gradual decrease in the particle size and layer number of MoSe_2_, which coincided with the characterization results of XRD. The content of glucose in the precursor also affected the exposed crystal planes of MoSe_2_ and Mo_2_C. Setting the C/Mo ratio in the precursor at 15 and 35, lattice fringes of 0.28 nm and 0.23 nm (Figure S2e,f) could be observed in the HRTEM images, which were attributed to the (100) of MoSe_2_ and the (121) of Mo_2_C [46,47]. However, lattice fringes of 0.26 nm and 0.21 nm assigned to the (102) of MoSe_2_ and the (112) of Mo_2_C were observed (Figure S2h) when the C/Mo ratio in the precursor rose to 75 [48]. Owing to the different crystal faces that would display different catalytic performance [49], it can be inferred that there will be a large difference in the catalytic performance of CO_2_ hydrogenation to form methanol.

#### 2.1.2. XPS Analysis of Catalysts

The surface chemical state of the catalyst was further analyzed by XPS. The XPS survey spectra shown in Figure S3 suggest that the surface elements of the samples were Mo, Se, C and O. 

**Figure 3:**
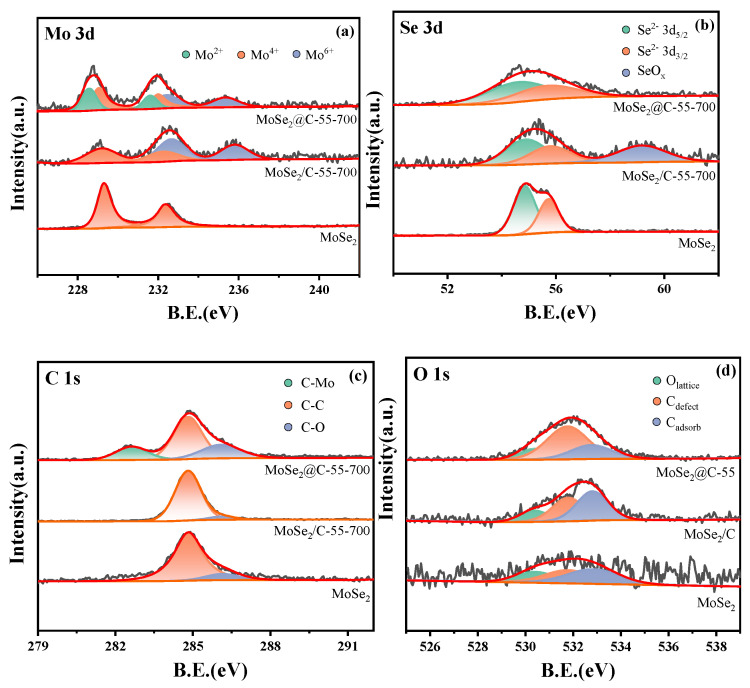
XPS spectra of MoSe_2_, MoSe_2_/C and MoSe_2_@C catalysts: (**a**) Mo 3d; (**b**) Se 3d; (**c**) C 1s; (**d**) O 1s.

The XPS spectra of the Mo 3d of the three samples (Figure 3a and Table S1) showed that characteristic peaks were observed at 229.1–229.3 eV and 232.1–232.3 eV, which were attributed to Mo^4+^ 3d_5/2_ and 3d_3/2_ in MoSe_2_ [48,50]. In addition, fitting peaks at 228.6 eV and 231.6 eV were also found in the Mo 3d of MoSe_2_@C-55, which corresponded to Mo^2+^ 3d_5/2_ and 3d_3/2_, indicating that Mo_2_C was generated during the preparation of MoSe_2_@C-55 [51,52], suggesting that a strong interaction between the carbon formed by the in situ carbonization of glucose and the Mo atoms was formed. The specific binding energy data in Table S1 show that the binding energy of Mo^4+^ 3d_5/2_ and 3d_3/2_ in MoSe_2_@C-55 was 0.2 eV lower than that of MoSe_2_ and MoSe_2_/C, which was caused by the electron transfer between MoSe_2_ and Mo_2_C [53], and a strong interaction was formed. In addition, peaks at binding energies of 232.5–232.7 eV and 235.4–235.7 eV, corresponding to Mo-O, were observed due to the surface oxidation that took place when MoSe_2_@C-55 and MoSe_2_/C were exposed to air [47,48,49,50,51,52], but the binding energy of MoSe_2_@C-55 was lower than that of MoSe_2_/C, which may be due to the protective effect of in situ-formed carbon on the surface of MoSe_2_@C-55, resulting in a reduction in the oxidation degree. This result demonstrates once again the robust interaction between carbon and Mo in MoSe_2_@C-55.

The Se 3d XPS spectra are displayed in Figure 3b, and two peaks at 54.7–54.8 eV and 55.7–55.8 eV (Table S2) were assigned to the Se^2-^ 3d_5/2_ and 3d_3/2_ of MoSe_2_ [42,46]. Moreover, MoSe_2_/C also showed a characteristic peak at about 59.1 eV, assigned to Se oxide (SeO_x_) [54]. In addition, as shown in Figure 3c and Table S3, the peaks at 284.8 eV and about 286.1 eV in the C 1s spectrum of all samples indicate the formation of a C-C bond and a C-O bond, respectively [55]. The high-resolution XPS spectra of O 1s are shown in Figure 3d and Table S4. The peaks at 530.4 eV, 531.8 eV and 532.8 eV indicate the formation of lattice oxygen (O_lat_), deficient oxygen (O_def_) and adsorbed oxygen (O_ads_), respectively [56,57]. The proportion of each oxygen species in all oxygen species is shown in Table S4. The proportion of O_def_ is the highest in the MoSe_2_@C-55 catalyst. The oxygen vacancies on the surface improved the dissociation of CO_2_ and tended to produce the desired methanol [57]. Moreover, only the MoSe_2_@C-55 sample showed a peak at 282.6 eV, which corresponded to a C-Mo bond [53,54]. The data listed in Figure S5 also show that the binding energy of Mo 3d showed a negative shift with the increase in the C/Mo ratio in the precursor, and the Mo^2+^/Mo^4+^ ratio on the surface of the catalyst displayed a positive correlation with the carbon content in the precursor (Table S5), suggesting that a higher carbon content in the precursor was beneficial to generating Mo_2_C. 

#### 2.1.3. Characterization of CO_2_ Adsorption Capacity

**Figure 4:**
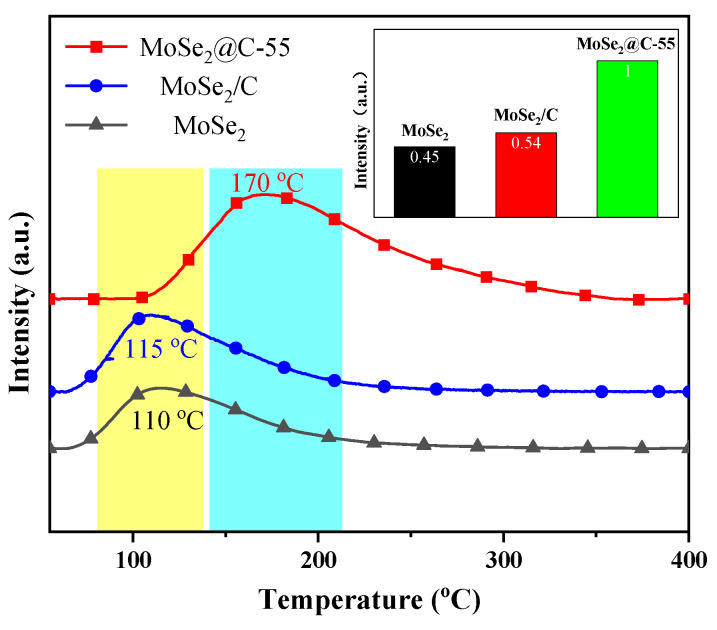
CO_2_-TPD profiles of MoSe_2_, MoSe_2_/C and MoSe_2_@C-55 catalysts.

The adsorptive capacity of a catalyst for CO_2_ is an important factor in its catalytic performance in CO_2_ hydrogenation. Therefore, CO_2_-TPD was carried out, and the desorption curves are displayed in Figure 4. According to the characterization results, all the CO_2_ desorption peaks of the prepared MoSe_2_, MoSe_2_/C and MoSe_2_@C-55 catalysts appeared at low temperatures, which corresponded to the presence of weak basic sites on the catalyst surface [58,59]. Compared with MoSe_2_ and MoSe_2_/C, MoSe_2_@C-55 showed the highest desorption temperature of CO_2_, which means that the activation degree of CO_2_ on the surface of MoSe_2_@C-55 was the highest, which would be beneficial to the hydrogenation of CO_2_. Because the magnitude of the peak area corresponds to the quantity of activated CO_2_ present on the catalyst surface, the relative desorbed amount of CO_2_ was calculated by calculating the area of these peaks, and the results are also inset in Figure 4. Based on the desorption amounts of CO_2_ from MoSe_2_@C-55 (the relative value is 100%), the desorption amounts of CO_2_ from the surface of MoSe_2_ and MoSe_2_/C were 45% and 54% (by calculating the area of the CO_2_ desorption peak). These results suggest that the order of CO_2_ adsorption amounts on the catalyst surface and the ability to activate CO_2_ were MoSe_2_@C-55>MoSe_2_/C>MoSe_2_. 

### 2.2. Catalytic Performance in CO_2_ Hydrogenation to Yield Methanol

In a fixed-bed reactor at 180 °C, 3MPa and a Gas Hourly Space Velocity (GHSV) of 3000 mL·g_cat_^−1^·h^−1^, the catalytic performance of MoSe_2_, MoSe_2_/C and MoSe_2_@C-55 was evaluated for CO_2_ hydrogenation to yield methanol (Table 1). In the presence of the MoSe_2_ catalyst, the conversion of CO_2_ was as low as 2.4%, and only a moderate selectivity (52.5%) of methanol was observed in the hydrogenation process, while CO was yielded with a selectivity of 33.1%. In this process, CO was generated from the reverse water gas shift (RWGS) reaction of CO_2_ on the surface of MoSe_2_, which was an important intermediate for synthesizing methanol. However, the insufficient active sites were exposed and could not achieve further efficient CO conversion, resulting in high CO selectivity [60]. At the same time, the CH_3_O^*^ intermediate generated by CO hydrogenation was more likely to undergo C-O cleavage to generate CH_3_^*^ and OH^*^ at the active sites, resulting in a high CH_4_ selectivity (10.2%) [32]. In addition, a small amount of dimethyl ether was generated by further dehydration of methanol, which was due to the weak acidity generated from the oxidation of the surface of MoSe_2_ [61]. The selectivity of methanol increased to 89.9% at a CO_2_ conversion of 4.3% over the MoSe_2_/C catalyst, prepared by using coconut shell carbon as support. This was attributed to an increase in the dispersion of MoSe_2_ on the support, which was beneficial for exposing more edge sites and surface defects due to the reduction in the particle size and layer number of MoSe_2_ [32]. The conversion of CO_2_ increased to 9.7% by employing MoSe_2_@C-55 as a catalyst prepared by using glucose as a carbon source with a C/Mo ratio of 55, while the selectivity of methanol further increased to 93.7% and the selectivity of CH_4_ decreased to 6.3%, which can be explained by the following reasons: On the one hand, the in situ-formed carbon gave a strong confinement to MoSe_2_, resulting in a decreasing layer number, which could expose more surface defects conducive to methanol generation. Moreover, the highest amount of deficient oxygen was detected by XPS, and the oxygen vacancies on the surface improved the dissociation of CO_2_ and tended to produce the desired methanol [57]. On the other hand, in situ-generated Mo_2_C interrupted the continuity of MoSe_2_, and the MoSe_2_-Mo_2_C heterojunction was formed, which was beneficial for exposing more active sites and activating H_2_ (Figure S5). The above factors all led to improved CO_2_ conversion and selectivity of methanol in CO_2_ hydrogenation. Moreover, the high desorption capacity and the high CO_2_ activation ability of MoSe_2_@C-55 were also important factors for increasing CO_2_ conversion. Based on the above research results, the catalytic performances of a series of MoSe_2_@C-x (x = 15, 35 and 75) catalysts with different C/Mo ratios in the precursors were also evaluated (Table 1). It can be seen that the selectivity of methanol gradually increased with the increase in C/Mo ratios. When MoSe_2_@C-75 was used as the catalyst, the selectivity of methanol reached a maximum of 94.3%. This was due to the fact that the number of layers of MoSe_2_ was reduced as the carbon content in the precursors increased, which was favorable for the exposure of surface defects and conducive to the formation of methanol. The conversion of CO_2_ reached its maximum when MoSe_2_@C-55 was used as the catalyst. This took place because MoSe_2_@C-55 had a stronger ability to adsorb and activate CO_2_ (Figure S6), causing an acceleration of CO_2_ conversion. 

Compared with the Cu/In_2_O_3_-, CuZnAl- and MoS_2_-based catalysts in the literature, MoSe_2_@C-55 showed better selectivity for methanol under low reaction temperatures, as shown in Table S7.

**Figure 5:**
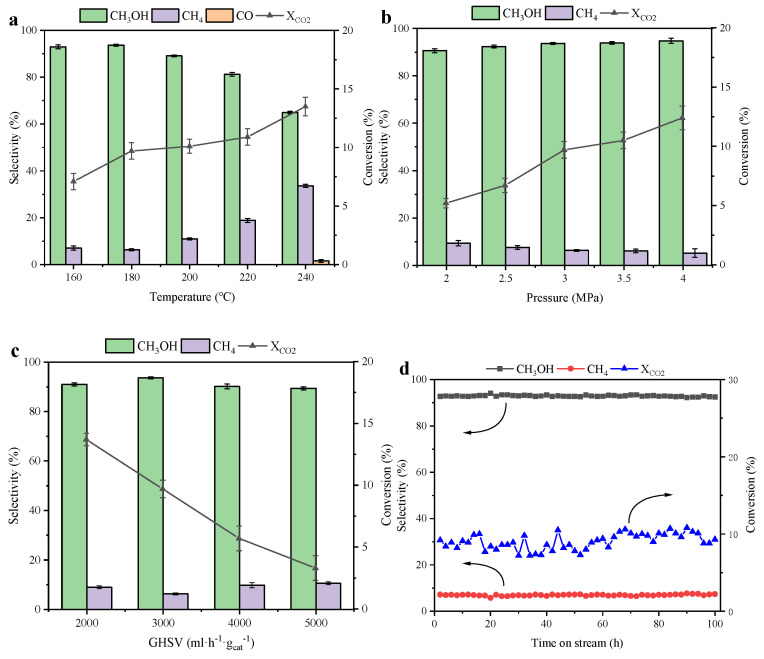
Catalytic performance of hydrogenation of CO_2_ over MoSe_2_@C-55 under different conditions: (**a**) effect of temperature; (**b**) effect of pressure; (**c**) effect of space velocity; (**d**) stability evaluation.

In CO_2_ hydrogenation, CO_2_ conversion and methanol selectivity were sensitive to the reaction temperature. Using MoSe_2_@C-55 as a catalyst, the effect of the reaction temperature was investigated under conditions of 3 MPa and a GHSV of 3000 mL·g_cat_^−1^·h^−1^ (Figure 5a). The results in Figure 5a show that the conversion of CO_2_ increased from 7.1% to 13.5% as the reaction temperature rose from 160 °C to 240 °C, suggesting that high temperatures are beneficial for CO_2_ to overcome the limitation of its thermodynamic stability, which accelerated the conversion rate of CO_2_ [62,63]. Setting the reaction temperature at 180 °C, the selectivity of methanol reached a maximum of 93.7% and gradually decreased as the reaction temperature increased. This took place because the reaction of CO_2_ hydrogenation to methanol was an exothermic reaction, and a higher temperature was not conducive to the formation of methanol. Since the reverse water gas reaction is an endothermic reaction [60], high temperatures are beneficial to its occurrence. Therefore, CO was not detected as a byproduct when the reaction temperature was not higher than 220 °C. But 1.5% of CO and 33.6% of CH_4_ were generated by raising the reaction temperature to 240 °C. As shown in Figure 5b, the influence of reaction pressure on the hydrogenation of CO_2_ was also investigated. The conversion of CO_2_ and the selectivity of methanol were all preferential to enhancing reaction pressure. When the reaction pressure was 2 MPa, CO_2_ conversion and methanol selectivity were both at a low level (5.6% and 90.6%, respectively). Moreover, the selectivity of methanol improved from 90.6 to 93.7% by changing the reaction pressure from 2 MPa to 3 MPa. When the reaction pressure was further increased, the selectivity of methanol did not change significantly. Finally, the effects of the space–time rate were also investigated (Figure 5c). With the increase in the space–time rate, the residence time of the feed gas on the surface of MoSe_2_@C-55 was shortened, resulting in a reduction in the conversion of CO_2_. When the space velocity was 3000 mL·g_cat_^−1^·h^−1^, the highest methanol selectivity at a moderate CO_2_ conversion was obtained. Under optimized reaction conditions, the test of the catalytic stability of the MoSe_2_@C-55 catalyst was performed, and the results are shown in Figure 5d. It indicated that the MoSe_2_@C-55 catalyst could be used continuously for at least 100 h without reducing catalytic activity and showing excellent catalytic stability. The selectivity of methanol was maintained at about 93.7% at a CO_2_ conversion of approximately 9.7%.

### 2.3. Reaction Mechanism

**Figure 6:**
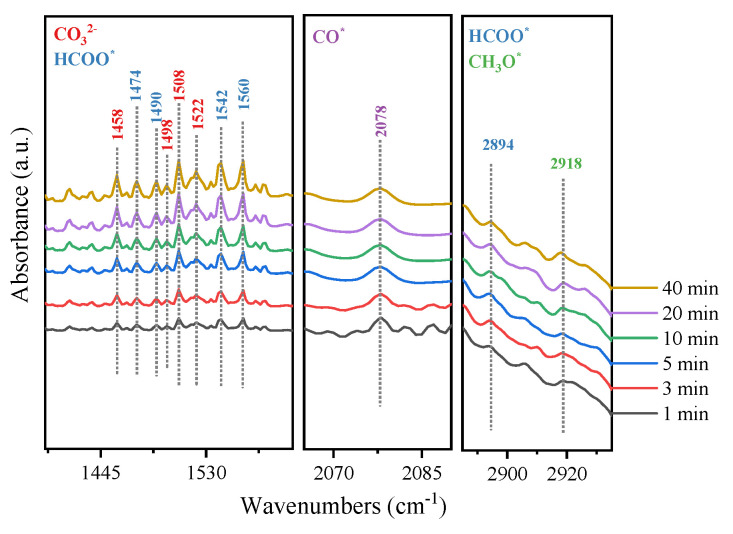
DRIFTS spectra of CO_2_ hydrogenation in the presence of MoSe_2_@C-55.

In order to explore the reaction mechanism of the hydrogenation of CO_2_ to generate methanol over the MoSe_2_@C-55 catalyst, the possible intermediates on the surface of MoSe_2_@C-55 were detected by in situ diffuse reflectance infrared Fourier transform spectroscopy (DRIFTS). Firstly, the catalyst was activated at 400 °C for 1 h in a pure H_2_ atmosphere and then refrigerated to 180 °C. Figure 6 shows the experimental results of the CO_2_ hydrogenation reaction stage at atmospheric pressure and a reaction temperature of 180 °C. 

**Scheme 1:**
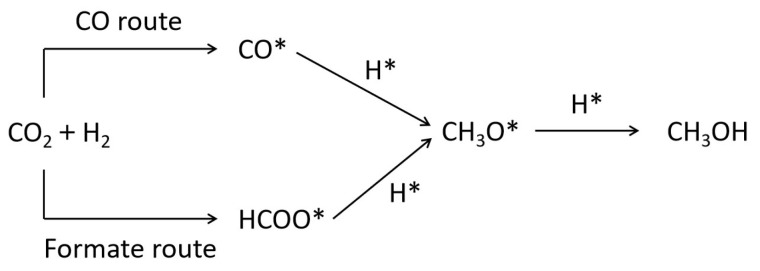
Supposed reaction path of CO_2_ hydrogenation to methanol in the presence of MoSe_2_@C-55.

From the in situ DRIFTS, two peaks were observed at 1458 and 1522 cm^−1^, corresponding to carbonate species (CO_3_^2−^) [64,65]. In addition, two peaks at 1498 and 1508 cm^−1^ were also observed, corresponding to monodentate and bidentate carbonate species [66], and their intensity gradually increased with the prolongation of the reaction time. As a result of these, CO_2_ first adsorbed on the surface of MoSe_2_@C-55 and then converted into a carbonate (CO_3_^2−^) species. The C-H bond vibration of the formate species at 1490 cm^−1^ [67] and spectral bands at 1560, 1542, 1474 and 2894 cm^−1^ could be observed, corresponding to the symmetric OCO stretching vibration and the stretching vibration of the formate species [68,69,70,71]. In addition, the characteristic absorption peak at 2918 cm^−1^ was attributed to the vibration of the C-H bond of the CH_3_O^*^ species [72]. The above results indicated that there may be a reaction path in the reaction process from CO_2_ hydrogenation to HCOO* species and finally to CH_3_OH. Specifically, the adsorbed CO_2_ was converted to carbonate, then HCOO* species were generated from the reaction between carbonate and hydrogen, and CH_3_O* was formed from the hydrogenation of the HCOO^∗^ species. Subsequently, CH_3_O* → CH_3_OH* → CH_3_OH (g) were performed. At the same time, a characteristic peak was observed at 2078 cm^−1^, attributed to CO^*^ [69], which provided evidence for the dissociation of CO_2_ when it was adsorbed on the catalyst surface. However, CO was not detected in the reaction products when the reaction was carried out at 180 °C, suggesting that the hydrogenation of CO occurred. Therefore, there is another reaction path in CO_2_ hydrogenation over the MoSe_2_@C-55 catalyst, where the adsorbed CO_2_ is dissociated to CO, which is then directly hydrogenated to CH_3_O^*^ and finally to CH_3_OH. The detailed reaction pathway is shown in Scheme 1.

## 3. Experiment

### 3.1. Materials

Ammonium molybdate ((NH_4_)_6_Mo_7_O_24_·4H_2_O, AMT) and glucose were obtained from Tianjin Kemiou Chemical Reagents Co., Ltd. (Tianjin, China). Selenium powder was purchased from MackLin Chemical Reagents Co., Ltd. (Shanghai, China), and sodium borohydride was obtained from Tianjin Kemiou Chemical Reagents Co., Ltd. (Tianjin, China). H_2_ (99.99%), N_2_ (99.99%), CO_2_ (99.99%) and 5% Ar/95% H_2_ (99.99%) were bought from Qinghua Gas Co., Ltd. (Harbin, China). Without further purification, all reagents were used directly.

### 3.2. Preparation of Catalysts

#### 3.2.1. Preparing the MoSe_2_@C-55 Catalyst

Ammonium molybdate (0.4022 g) and glucose (4.1381 g) (n(C)/n(Mo) = 55) were dissolved in deionized water. After removal of the water, the prepared product was dried at 110 °C for 12 h. Subsequently, it was mixed with selenium powder (0.3598 g) and placed in a tubular furnace. In a N_2_ atmosphere, the temperature was increased to 700 °C at a rate of 5 °C/min for 4 h, and MoSe_2_ confined in carbon material catalyst (MoSe_2_@C-55, where 55 is the carbon–molybdenum ratio (C/Mo) in the precursor) was obtained. When the C/Mo ratio in the precursors changed to 15, 35 and 75, they were named MoSe_2_@-15, MoSe_2_@-35 and MoSe_2_@-75, respectively.

#### 3.2.2. Preparing the MoSe_2_/C Catalyst

A total of 0.4022 g ammonium molybdate was impregnated into 1.5036 g coconut shell carbon by the impregnation method and dried at 110 °C overnight. After mixing the dried solid and 0.3598 g selenium powder, the resulting products were roasted in a tube furnace. In a H_2_/N_2_ atmosphere containing 30% H_2_, the temperature was raised to 700 °C at 5 °C/min for 4 h. The MoSe_2_/C sample was obtained.

#### 3.2.3. Preparing the MoSe_2_ Catalyst

An aqueous solution containing sodium borohydride (0.3972 g) was added drop by drop to 0.5527 g selenium powder, and the mixture was stirred to form a reddish-brown solution at 70 °C. Then, the reddish-brown mixture was added to an aqueous solution containing ammonium molybdate (0.6180 g). After stirring at ambient temperature for 30 min, the mixture was put into a 50 mL stainless steel autoclave lined with PTFE and crystallized at 220 °C for 24 h, then cooled to ambient temperature. Subsequently, it was centrifuged, washed, dried and put into a tubular furnace. In a N_2_ atmosphere, the temperature of the tubular furnace rose to 550 °C at a rate of 5 °C/min, and it was held for 2 h. Then, it was refrigerated to ambient temperature, and the MoSe_2_ sample was obtained.

### 3.3. Characterization

X-ray powder diffraction (XRD, Bruker D8, Advance, Salbrücken, Germany) of Cu-Kα radiation diffraction (ƛ = 1.5406A) was used to characterize the crystal structure of the samples, and transmission electron microscopy (TEM, JEM-2100, Tokyo, Japan) and high-resolution transmission electron microscopy (HRTEM, JEM-2100, Tokyo, Japan) were used to observe their morphology and size. An X-ray photoelectron spectrometer (ESCALAB 250-11 OOV, Thermo Fisher, Waltham, MA, USA) was employed to determine the types and valence of the elements on their surface. CO_2_ temperature-programmed desorption (TPD) was carried out on a chemisorption instrument equipped with a thermal conductivity detector (TCD). The process was as follows: After pretreatment at 500 °C for 60 min in Ar (40 mL/min), the sample (0.2 g) was exposed to CO_2_ (40 mL/min) for 60 min while it was cooled to 50 °C. Subsequently, the physically adsorbed CO_2_ was eliminated in a pure Ar (40 mL/min) atmosphere for 60 min. Then, the desorption of CO_2_ was measured from 50 °C to 500 °C at a rate of 10 °C/min in an Ar (40 mL/min) atmosphere. On a Perkin Elmer frontier spectrometer (Waltham, MA, USA), in situ diffuse infrared Fourier transform spectroscopy (DRIFTS) measurements were carried out. Firstly, the sample was pretreated at 400 °C for 60 min with a H_2_ flow of 30 mL/min and then chilled to 180 °C. Subsequently, gas feed (H_2_ 30 mL/min and CO_2_ 10 mL/min) was introduced after scanning the background spectrum in the range of 400–4000 cm^−1^, and the samples were scanned four times in the first 10 min and twice in 30 min.

### 3.4. Catalytic Performance Evaluation

**Figure 7:**
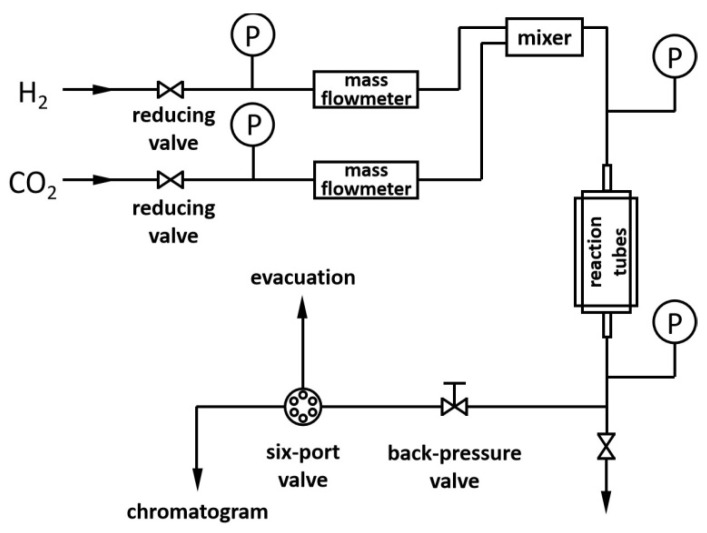
Schematic diagram of the reaction device.

The hydrogenation of CO_2_ to yield methanol was performed in a fixed-bed reactor (inner diameter of 6 mm). The schematic diagram of the reaction device is shown in Figure 7. After the catalyst (0.5 g) was pretreated in pure H_2_ at 400 °C for 3 h and chilled to 180 °C, the feed gas (H_2_/CO_2_ ratio of 3/1) was introduced into the reactor under 3.0 MPa. The reaction tail gas was quantitatively analyzed using a gas chromatograph equipped with a hydrogen flame detector (GC-7900, Tianmei, Shanghai, China) and a thermal conductivity detector (GC-7890II, Tianmei, Shanghai, China).

## 4. Conclusions

MoSe_2_ catalysts were prepared by different loading methods and applied to carry out the hydrogenation of CO_2_ to methanol. The introduction of carbon materials can effectively improve the dispersion of MoSe_2_. When glucose was introduced into the precursor as a carbon source, small particles and a few layers of MoSe_2_ were formed in the MoSe_2_@C-55 catalyst under the confinement of the in situ-formed carbon material, which was beneficial for exposing more active sites.

Due to the strong interaction between MoSe_2_ and the in situ-formed carbon, the MoSe_2_-Mo_2_C heterojunction was generated, which interrupted the continuity of MoSe_2_. Under the confinement effect of the in situ-formed carbon material, the layer number and particle size of MoSe_2_ were reduced in the MoSe_2_@C-55 catalyst, which was conducive to exposing more active sites.

Over the MoSe_2_@C-55 catalyst, a higher methanol selectivity of 93.7% and excellent catalytic stability in the continuous reaction within 100 h provide a prospect for the application of the high-efficiency catalyst under the cooperation of the MoSe_2_-Mo_2_C heterojunction and the in situ-formed carbon material. The results of the in situ DRIFTS proved the HCOO* and CO* species were the intermediates for synthesizing methanol from CO_2_ in the presence of a MoSe_2_@C-55 catalyst.

## Figures and Tables

**Table 1:** Results of CO_2_ hydrogenation to methanol over different catalysts ^a^.

Catalyst	Conversion (%)	Selectivity (%)			
		CH_3_OH	CH_4_	CO	CH_3_OCH_3_
MoSe_2_	2.4	52.5	10.2	33.1	4.2
MoSe_2_/C	4.3	89.9	10.1	-	-
MoSe_2_@C-55	9.7	93.7	6.3	-	-
MoSe_2_@C-15	2.8	72.3	27.7	-	-
MoSe_2_@C-35	3.3	89.5	10.5	-	-
MoSe_2_@C-75	4.4	94.3	5.7	-	-

^a^ Reaction conditions: 180 °C, 3 MPa, GHSV 3000 mL·g_cat_^−1^·h^−1^, H_2_:CO_2_ = 3.

## Data Availability

All data generated or analyzed during this study are included in this published article.

## Supplementary Materials

The following supporting information can be downloaded at https://www.mdpi.com/article/10.3390/molecules29102186/s1: Figure S1. XRD patterns of MoSe_2_@C-x catalysts prepared with different C/Mo ratios; Figure S2. TEM and HRTEM images of MoSe_2_@C-x catalysts prepared with different C/Mo ratios: (a,e) MoSe_2_@C-15; (b,f) MoSe_2_@C-35; (c,g) MoSe_2_@C-55; (d,h) MoSe_2_@C-75; Figure S3. Survey XPS spectra of MoSe_2_, MoSe_2_/C and MoSe_2_@C catalysts; Figure S4. XPS spectra of MoSe_2_@C-x catalysts prepared with different C/Mo ratios: (a) survey spectrum; (b) Mo 3d; (c) Se 3d; (d) C 1s; (e) O 1s; Figure S5. Activating H_2_ on the MoSe_2_-Mo_2_C heterojunction; Figure S6. CO2-TPD of MoSe_2_@C-x with different C/Mo ratios; Table S1. The valence state and distribution of the Mo element on the surface of the MoSe_2_, MoSe_2_/C and MoSe_2_@C catalysts; Table S2. The valence state and distribution of the Se element on the surface of the MoSe_2_, MoSe_2_/C and MoSe_2_@C catalysts; Table S3. The valence state and distribution of the C element on the surface of the MoSe_2_, MoSe_2_/C and MoSe_2_@C catalysts; Table S4. The valence state and distribution of the O element on the surface of the MoSe_2_, MoSe_2_/C and MoSe_2_@C catalysts; Table S5. The valence state and distribution of the Mo element on the surface of MoSe_2_@C-x; Table S6. The valence state and distribution of the O element on the surface of MoSe_2_@C-x; Table S7. Comparison betweenMoSe_2_@C-55 and reported catalysts in CO_2_ hydrogenation to methanol [73,74,75,76,77].
