# Supplementary material for: In Situ Carbon-Confined MoSe2 Catalyst with Heterojunction for Highly Selective CO2 Hydrogenation to Methanol
Source: Molecules. 2024 May 8;29(10):2186. doi: 10.3390/molecules29102186 (PMC11123706; doi:10.3390/molecules29102186)
Supplement: Supplementary file 1 [file molecules-29-02186-s001.zip › molecules-2984566-supplementary.pdf]

In Situ Carbon-Confined $MoSe_2$ Catalyst with Heterojunction for Highly Selective CO_2 Hydrogenation to Methanol

Yanyang Sun, Linfei Xiao * and Wei Wu *

National Center for International Research on Catalytic Technology, School of Chemistry and Material Sciences, Heilongjiang University, Harbin 150080, China; sunyanyang99@163.com
* Correspondence: xiaolf@hlju.edu.cn (L.X.); wuwei@hlju.edu.cn (W.W.)

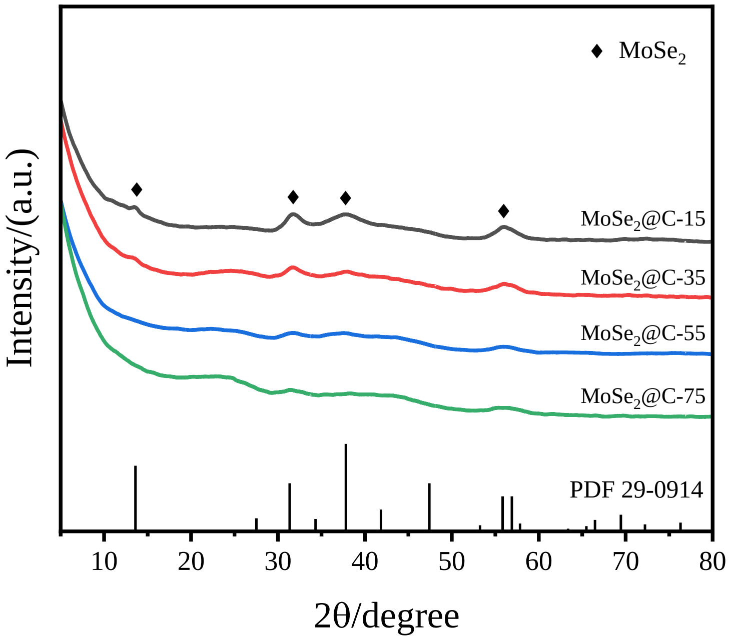

Figure S1. XRD patterns of $MoSe_2$@C-x catalysts prepared with different C/Mo ratios.

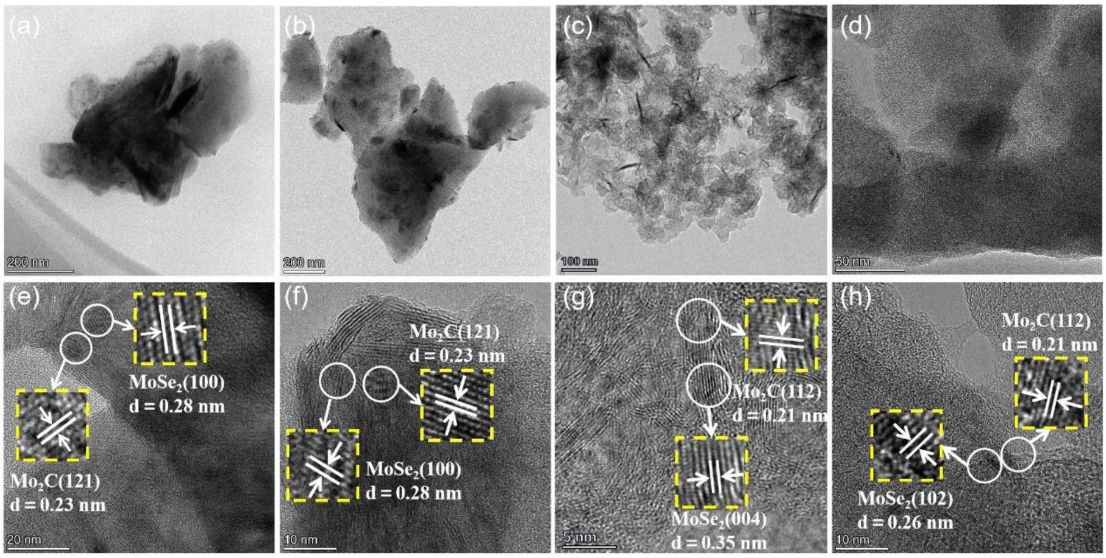

Figure S2. TEM and HRTEM images of $MoSe_2$@C-x catalysts prepared with

different C/Mo ratios: (a, e) $MoSe_2$@C-15; (b, f) $MoSe_2$@C-35; (c, g) $MoSe_2$@C-55; (d, h) $MoSe_2$@C-75.

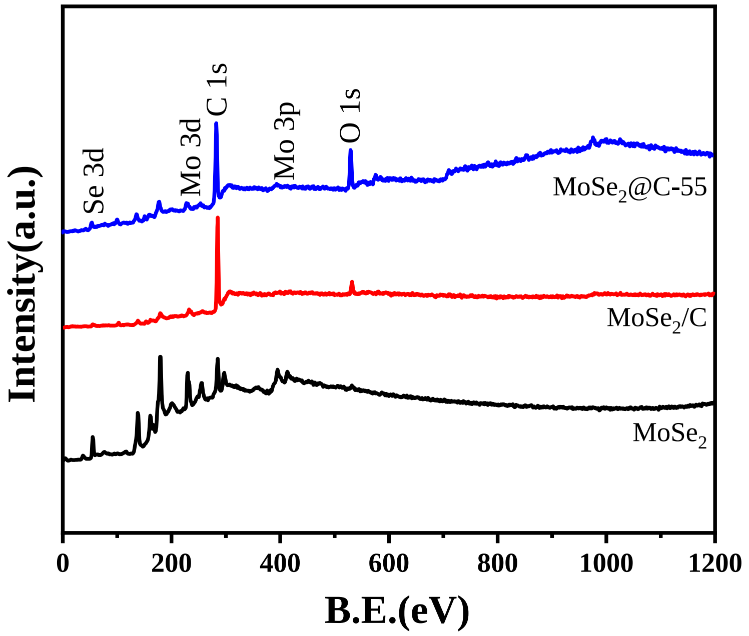

Figure S3. Survey XPS spectra of $MoSe_2$, $MoSe_2$/C and $MoSe_2$@C catalysts

Table S1. The valence state and distribution of Mo element on the surface of $MoSe_2$, $MoSe_2$/C and $MoSe_2$@C catalysts

Catalysts	Mo^{2+} $3d_{5/2}$	Mo^{2+} $3d_{3/2}$	Mo^{4+} $3d_{5/2}$	Mo^{4+} $3d_{3/2}$	Mo^{6+} $3d_{5/2}$	Mo^{6+} $3d_{3/2}$
$MoSe_2$	-	-	229.3	232.3	-	-
$MoSe_2$/C			229.2	232.3	232.7	235.7
$MoSe_2$@C-55	228.6	231.6	229.1	232.1	232.5	235.4

Table S2. The valence state and distribution of Se element on the surface of $MoSe_2$, $MoSe_2$/C and $MoSe_2$@C catalysts.

Catalysts	Se^{2-} $3d_{5/2}$	Se^{2-} $3d_{3/2}$	SeO_x
$MoSe_2$	54.8	55.7	-
$MoSe_2$/C	54.8	55.8	59.1
$MoSe_2$@C-55	54.7	55.8	-

Table S3. The valence state and distribution of C element on the surface of $MoSe_2$, $MoSe_2$/C and $MoSe_2$@C catalysts.

Catalysts	C-Mo	C-C	C-O
$MoSe_2$	-	284.8	286.1
$MoSe_2$/C	-	284.8	286.1
$MoSe_2$@C-55	282.6	284.8	286.1

Table S4. The valence state and distribution of O element on the surface of $MoSe_2$, $MoSe_2$/C and $MoSe_2$@C catalysts

Catalysts	Lattice oxygen	Deficient oxygen	Adsorbed oxygen
$MoSe_2$	530.4(23.3%)	531.8(34.6%)	532.8(42.1%)
$MoSe_2$/C	530.4(16.4%)	531.8(37.4%)	532.8(46.2%)
$MoSe_2$@C-55	530.4(15.1%)	531.8(59.6%)	532.8(25.3%)

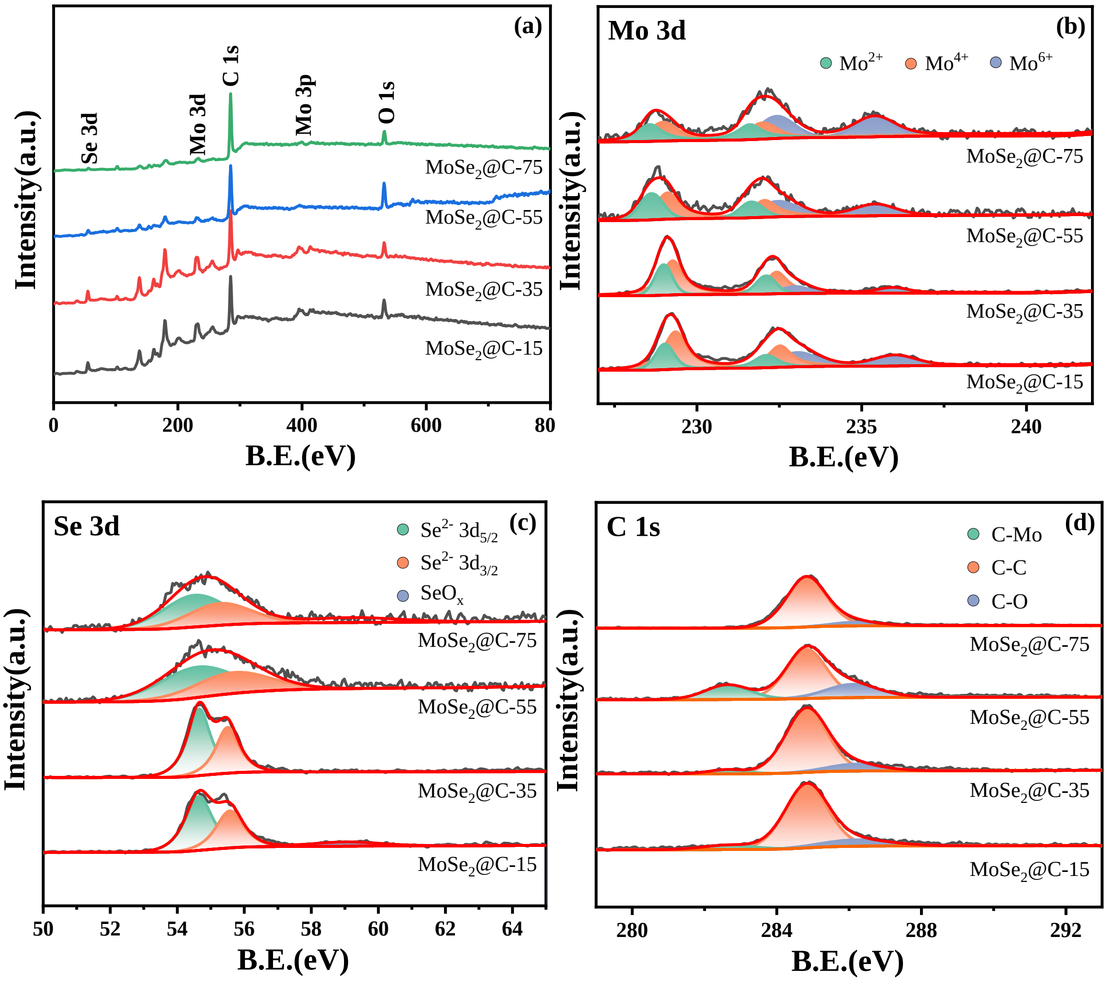

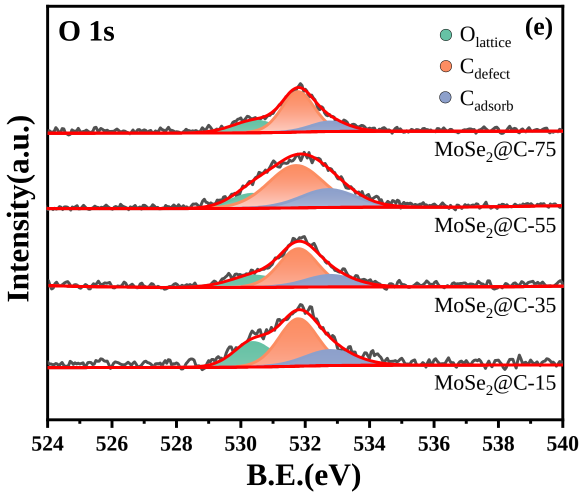

Figure S4. XPS spectra of $MoSe_2$@C-x catalysts prepared with different C/Mo ratios: (a) survey spectrum; (b) Mo 3d; (c) Se 3d; (d) C 1s; (e) O 1s.

Table S5. The valence state and distribution of Mo element on the surface of $MoSe_2$@C-x

Catalysts	Mo $3d_{3/2}$ (eV)			
	Mo^{2+}	Mo^{4+}	Mo^{6+}	Mo^{2+}/ Mo^{4+}
$MoSe_2$@C-15	232.1(26.6%)	232.5 (48.4%)	236.0 (25.0%)	0.55
$MoSe_2$@C-35	232.0 (33.2%)	232.4 (56.3%)	235.9 (17.5%)	0.59
$MoSe_2$@C-55	231.6 (28.9%)	232.1(43.6%)	235.3(28.9%)	0.66
$MoSe_2$@C-75	231.6 (27.4%)	232.1(37.5%)	235.4(35.2%)	0.73

Table S6. The valence state and distribution of O element on the surface of $MoSe_2$@C-x

Catalysts	lattice oxygen	deficient oxygen	adsorbed oxygen
$MoSe_2$@C-15	530.4(26.8%)	531.8(51.8%)	532.8(21.4%)
$MoSe_2$@C-35	530.4(21.2%)	531.8(57.1%)	532.8(21.7%)
$MoSe_2$@C-55	530.4(15.1%)	531.8(59.6%)	532.8(25.3%)
$MoSe_2$@C-75	530.4(23.3%)	531.8(60.1%)	532.8(16.6%)

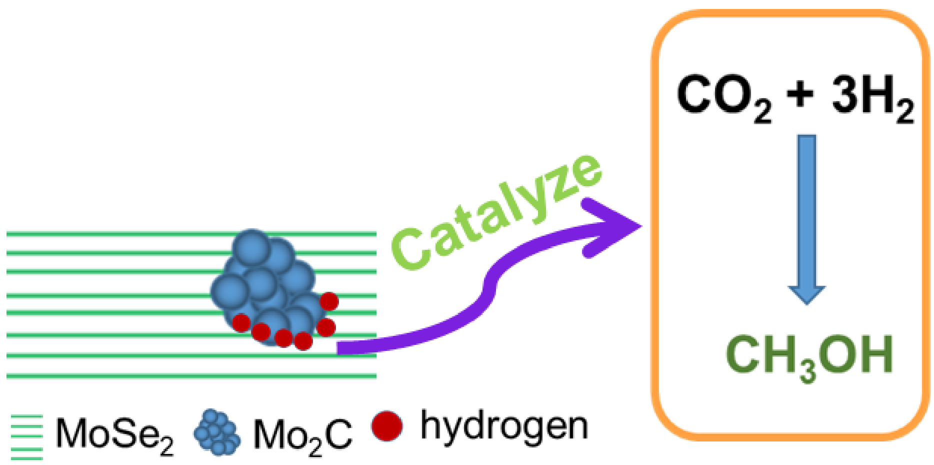

Figure S5. Activating H_2 on the $MoSe_2$-Mo_2C heterojunction

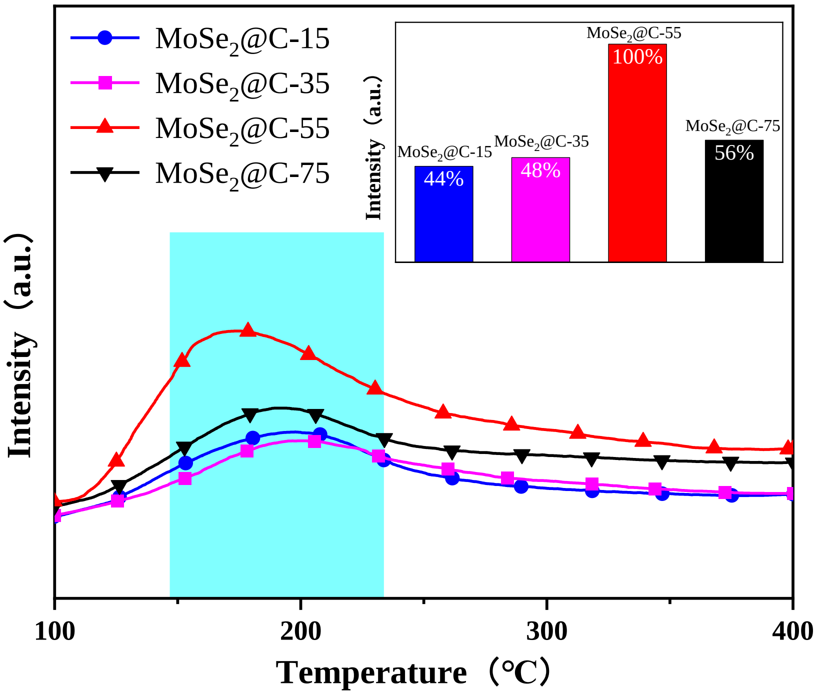

Figure S6. CO_2-TPD of $MoSe_2$@C-x with different C/Mo ratios

Table S7. Comparison of the $MoSe_2$@C-55 with the reported catalysts in the CO_2 hydrogenation to methanol.

Catalyst	Reaction conditions	Conv. (%)	Sel. (%)	Ref.
CNTs@MoS_2	260 °C,5 MPa, 24000 ml·g_{cat}^{-1}·h^{-1},H_2/CO_2=3	7.5	78.1	[73]
Cu/MoS_2@SiO_2	260 °C,5 Mpa, 24000 ml·g_{cat}^{-1}·h^{-1},H_2/CO_2=4	9.0	72.5	[74]
MoS_2/$Ni_{0.2}$	260 °C,5 Mpa, 12000 ml·g_{cat}^{-1}·h^{-1},H_2/CO_2=3	1.0	83.7	[75]
5%Cu-MoS2	220 °C,5 MPa, 12000 ml·g_{cat}^{-1}·h^{-1},H_2/CO_2=4	5.4	86.0	[60]

h-MoS_2/ZnS	260 ^{o}C,5 MPa, 15000 ml·g_{cat}^{-1}·h^{-1},H_2/CO_2=4	9.0	67.3	[69]
Cu/In_2O_3	300 ^{o}C,5 MPa, 6000 ml·g_{cat}^{-1}·h^{-1},H_2/CO_2=4	18.4	44.4	[76]
Cu/ZnO/Al_2O_3/ZrO_2	250 ^{o}C,5 MPa, 20000 ml·g_{cat}^{-1}·h^{-1},H_2/CO_2=3	20.2	44.9	[77]
$MoSe_2$@C-55	180 ^{o}C,3 MPa, 3000 ml·g_{cat}^{-1}·h^{-1},H_2/CO_2=3	9.7	93.7	This work
